# Does emotional intelligence have the same role in each risk behaviour?

**DOI:** 10.1192/j.eurpsy.2022.1773

**Published:** 2022-09-01

**Authors:** M.T. Sánchez-López, P. Fernández-Berrocal, R. Gómez-Leal, A. Megías-Robles

**Affiliations:** Faculty of Psychology. University of Málaga, Department Of Basic Psychology, Málaga, Spain

**Keywords:** Emotional Intelligence, Gender differences, risk-taking, risk domain

## Abstract

**Introduction:**

One of the most important factors that represents a threating both physical and psychological health in our lives is the individual’s risk behaviour. Though emotions exert a strong influence on risk decision-making, the literature studying the role of emotional abilities on the tendency to engage in risk behaviour is scarce.

**Objectives:**

The aim was to explore the relationship between emotional intelligence (Attention, Clarity, and Repair) and risk behaviour in its different domains (Ethical, Health, Financial, Social, and Recreational domains). We also examined whether there were gender differences in both variables.

**Methods:**

A Spanish community sample of 1435 participants (M_age_ = 29.84, ranging from 18 to 70 years old; 61.9% women) were assessed in levels of EI and risk-taking by the TMMS-24 and DOSPERT-30 scales.

**Results:**

The result revelated that emotional intelligence was positive related with Social and Recreational domains, and negative related with Ethical and Health domains. Moreover, women showed higher scores for EI and Social risk-taking domain than men, and men showed higher scores for Ethical, Financial, Health, and Recreational risk-taking domains.

**Conclusions:**

These findings show and support that EI is differentially related to risk behaviour depending on the risk domain studied. We suggest that higher levels of EI could be adaptive for risk behaviour regardless the directionality of the relationship. Considering the impact of health-related risky behaviours on public health and individual well-being, the development of effective risk prevention programs that train emotional abilities could reduce the incidence of these behaviours in our society.

**Disclosure:**

No significant relationships.

